# The Conventional Cost‐Effectiveness and Distributional Cost‐Effectiveness of Semaglutide for Obesity Treatment

**DOI:** 10.1002/oby.70311

**Published:** 2026-10-07

**Authors:** Neeti S. Kulkarni, David Hutton, Corwin Rhyan, Jeffrey S. McCullough, Michaela Greenlee, Ankur Pandya

**Affiliations:** ^1^ Department of Health Management and Policy University of Michigan School of Public Health Ann Arbor Michigan USA; ^2^ Department of Global Health and Population Harvard T.H. Chan School of Public Health Boston Massachusetts USA; ^3^ Department of Health Policy and Management Harvard T.H. Chan School of Public Health Boston Massachusetts USA

**Keywords:** economic evaluation, equity‐based research, GLP‐1 agonists

## Abstract

**Objective:**

This study aimed to evaluate the conventional and distributional cost‐effectiveness of semaglutide, laparoscopic sleeve gastrectomy (LSG), and a “do nothing” option in Black and White racial subgroups.

**Methods:**

A Markov simulation model was developed from a healthcare perspective over a 25‐year time horizon for Black and White adults, aged 65–70, with BMI ≥ 35 and cardiovascular disease. Three interventions were modeled: semaglutide 2.4 mg, LSG, and “do nothing.”

**Results:**

Semaglutide and LSG interventions are cost‐effective compared to a “do nothing” strategy (ICERs = $7300/QALY and $12,100/QALY, respectively). Compared to semaglutide, LSG is more expensive but cost‐effective (ICER = $53,700/QALY). Semaglutide is more equitable but is not distributionally cost‐effective compared to LSG, because it has a lower EDE within our defined inequality aversion (*ε*) range. Across 10,000 probabilistic iterations, LSG has a 57% probability and semaglutide has a 43% probability of being the most cost‐effective at the willingness‐to‐pay threshold of $100,000/QALY. Semaglutide becomes distributionally cost‐effective when *ε* ≥ 14.72, where equity impacts of semaglutide outweigh the conventional cost‐effectiveness outcomes.

**Conclusions:**

LSG is the most conventionally cost‐effective treatment option with some uncertainty. Semaglutide is marginally more equitable than LSG but is not a distributionally cost‐effective strategy.

## Study Importance

- What is already known?
  ○ At its previous price, semaglutide was cost‐ineffective or dominated compared to other treatment alternatives for obesity in the general population.
- What does this study add?
  ○ Semaglutide is cost‐effective compared to “doing nothing” in our study population, but laparoscopic sleeve gastrectomy (LSG) surgery is likely more cost‐effective than semaglutide.
  ○ Semaglutide is a marginally more equitable intervention than LSG surgery but is likely not distributionally cost‐effective.
- How might these results change the direction of research or the focus of clinical practice?
  ○ Both treatments should be available to Medicare patients with at least Class II obesity and cardiovascular disease.
  ○ Despite worse surgical outcomes for Black patients, LSG surgery is still an effective procedure for both Black and White Medicare subpopulations.

## 1 Introduction

GLP‐1 agonists for obesity treatment have imposed a substantial cost burden on the U.S. healthcare system; in response, the Trump administration has negotiated with the two main manufacturers (Novo Nordisk and Eli Lilly) to lower prices significantly. Under their agreement, Medicare and Medicaid prices decrease to $245 per month with a $50 patient copay [1]. Medicare has chosen to implement these price changes with the GLP‐1 Bridge pilot program, launched on July 1, 2026, until the end of 2027 [2]. This initiative represents a major policy shift in the current obesity treatment landscape. While GLP‐1 agonists, such as semaglutide (Ozempic and Wegovy), have received widespread interest in recent years, their high prices have led to limited access for patients. Several conventional cost‐effectiveness analyses (CEA) have found that, at original branded list prices, semaglutide was cost‐ineffective or dominated (i.e., less effective, more costly) compared to bariatric surgery or other lifestyle modifications [3, 4, 5]. A more recent analysis in 2025 found some favorable evidence for the economic efficiency of semaglutide compared to lifestyle modifications using net prices [6]. However, none of these analyses has been updated since the recent GLP‐1 agonist price reductions.

There remains an urgent need to address the current obesity epidemic in economic evaluations. Obesity prevalence among older adults in the U.S. is nearly 40% and is costly—the U.S. spends approximately $173 billion annually on medical expenses related to obesity [7]. In addition to its economic impact, obesity also has pronounced racial differences. Among Medicare fee‐for‐service beneficiaries, Black individuals have the highest rate of obesity at 24%, followed by White individuals at 19% [8, 9]. As the obesity epidemic continues, it is crucial to identify both efficient and equitable interventions for obesity treatment.

Bariatric surgery is an established and effective procedure for obesity [10]. However, racial disparities in bariatric surgery are well documented, specifically regarding differential outcomes between Black and White patients. Black bariatric surgery patients tend to experience less weight loss than their White counterparts. Furthermore, Black patients have a higher rate of complications and healthcare resource utilization after surgery [11, 12, 13]. Given the already existing racial disparities in obesity, the marked difference in surgical outcomes between Black and White patients may plausibly worsen the disparities further.

Medicare has approved coverage of semaglutide 2.4 mg (Wegovy) for individuals with overweight or obesity and an established cardiovascular condition since April 2024 [14]. Unlike bariatric surgery, the efficacy and side effects associated with semaglutide appear to be more similar across Black and White patients [15]. Current economic evaluations of obesity treatments have not considered subgroups such as the Black population that experience heterogeneous outcomes, which may lead to increased health disparities [16]. Thus, the objective of this study is to incorporate the newly negotiated price of semaglutide in both a conventional CEA and a distributional CEA in Black and White subgroups for semaglutide compared to laparoscopic sleeve gastrectomy (LSG) surgery, the most common form of bariatric surgery. Distributional cost‐effectiveness analysis (DCEA) is an extension of a conventional CEA that also incorporates an intervention's impact on population health disparities [17]. Given that there may be low uptake to either surgical or pharmacological obesity interventions, we additionally compare both semaglutide and LSG to a “do nothing” option [18].

## 2 Methods

### 2.1 Model Overview

We developed a 25‐year state transition Markov cohort model with a cycle length of 1 month to simulate three possible interventions: “do nothing,” injectable semaglutide 2.4 mg (Wegovy), and LSG surgery. The model assumes a healthcare system perspective, which includes patient, provider, and payer costs, such as the direct costs of treatment, as well as the broader medical costs of living with obesity for patients. The model begins after the monthly price of semaglutide has dropped to $245. Although individuals with overweight or obesity and a cardiovascular condition are eligible to receive semaglutide, only individuals with an obesity‐related comorbidity (such as a cardiovascular disease) who have unsuccessfully attempted lifestyle modifications and are in obesity Class II or higher (BMI ≥ 35) are eligible to receive both semaglutide and LSG under Medicare regulations. Therefore, the study population may start in obesity Class II (BMI 35–40), obesity Class III.I (BMI 40–50), or obesity Class III.II (BMI ≥ 50) health states (Figure 1). Individuals were assumed to start at the median BMI for their initial health state. Additionally, although Medicare does not impose a formal upper age limit for bariatric surgery, the starting population was restricted to individuals aged 65–70 years. Clinical opinion has confirmed that patients with Class II obesity and cardiovascular disease above the age of 70 would likely be at too high a risk for surgery. We estimated populations of approximately 14,000 and 86,000 individuals in the Black and White subgroups, respectively. To focus on maximum potential weight loss, the model excludes patients with diabetes. The model does not include other bariatric surgeries besides LSG surgery. Although Roux‐en‐Y gastric bypass (RYGB) surgery is another established form of bariatric surgery, LSG surgeries are now much more commonly performed in the U.S. than RYGB surgeries and are therefore a more realistic treatment alternative [19]. Lifestyle modifications are also not included as an intervention in the model because for Medicare beneficiaries to qualify for bariatric surgery they must have already unsuccessfully attempted lifestyle modifications [20]. Furthermore, literature and clinical opinion have confirmed that adherence to lifestyle modifications for older adults with cardiovascular disease tends to be very low [21]. Therefore, we characterize the strategy in which the study population has tried and failed at lifestyle modifications but has not received semaglutide or LSG surgery as the “do nothing” strategy.

**FIGURE 1:**
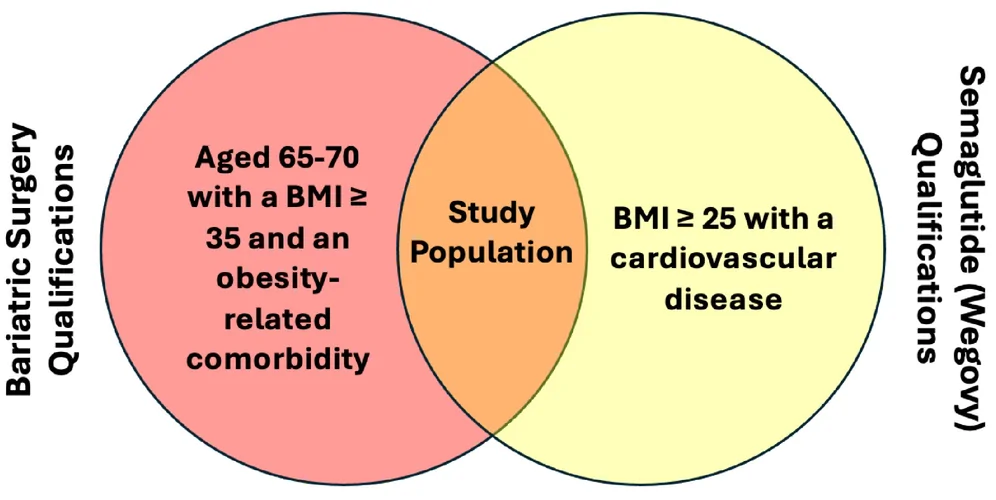
Venn diagram that depicts criteria to qualify for both semaglutide and laparoscopic sleeve gastrectomy (LSG) interventions under the current Medicare indications, where the intersection of the two circles is the study population. [Color figure can be viewed at wileyonlinelibrary.com]

In the simulation model, under the “do nothing” strategy, individuals, on average, gain a small amount of weight each month. Each cycle, they may remain in their current health state, move to higher obesity class states, or die. Under the semaglutide intervention, individuals, on average, lose weight each month in their first 16 months of the intervention and then remain at that weight for the duration of the model or until death. Under the LSG intervention, on average, individuals lose weight in their first year of treatment, and then slowly regain a minimal amount of weight each month thereafter for the duration of the model or until death. Figure 2 shows a diagram of all health states in the model. The model was run separately for the Black and White study populations. A weighted average of the results was calculated to obtain results for the overall population for the conventional CEA.

**FIGURE 2:**
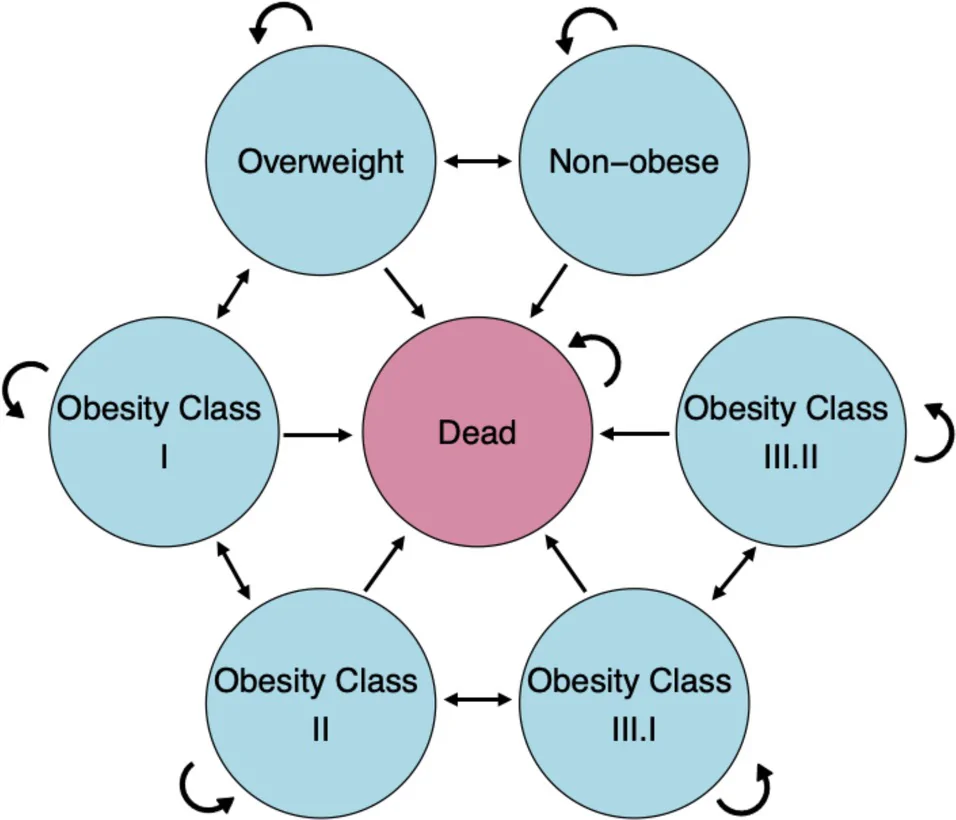
Model diagram depicting seven possible health states: non‐obese (BMI < 25), overweight (25 ≤ BMI < 30), obesity Class I (30 ≤ BMI < 35), obesity Class II (35 ≤ BMI < 40), obesity Class III.I (40 ≤ BMI < 50), obesity Class III.II (BMI ≥ 50), and dead. [Color figure can be viewed at wileyonlinelibrary.com]

This study did not require institutional review board approval as all analyses were based on publicly available data. This study was reported in alignment with the Consolidated Health Economic Evaluation Reporting Standards (CHEERS) reporting guideline for economic analyses [22].

### 2.2 Parameter Estimation

Parameter estimates obtained from the literature were generally assumed to be for the White adult population, since White individuals represent the majority of study populations. If Black‐specific, cardiovascular disease‐specific, and age‐specific estimates could not be obtained directly, we used multipliers, odds ratios, and risk ratios obtained from the literature to adjust the original parameters to fit the study populations. Treatment effects were obtained from clinical trials and modified as needed. Key base‐case parameter values and their ranges are listed in Table 1. All parameter values, as well as the assumptions and calculations used to derive them, can be found in the online Supporting Information.

**TABLE 1:** Select model parameters for weight trajectories, intervention costs, and complication rates.

Parameter	White population	Black population	Source
Weight trajectories			
“Do nothing” BMI points gained/month	0.024 (0, 0.026)		Zajacova et al. [23]
Semaglutide BMI points change/month (Year 1)	−0.26 (−0.24, −0.28)		Wilding et al. [24]; Ryan et al. [25]
LSG BMI points change/month (Months 0–6)	−1.62 (−1.30, −1.93)	−1.36 (−1.09, −1.62)	Salminen et al. [26]; Fernández‐Ananín et al. [27]; Wood et al. [13]
LSG BMI points change/month (Months 6–12)	−0.20 (−0.52, 0.13)	−0.17 (−0.44, 0.11)	Salminen et al. [26]; Fernández‐Ananín et al. [27]; Wood et al. [13]
LSG BMI points change/month (Years 2–5)	0.04 (−0.004, 0.09)		Salminen et al. [26]; Fernández‐Ananín et al. [27]; Wood et al. [13]
LSG BMI points change/month (Years 6–25)	0.02 (−0.02, 0.06)		Salminen et al. [26]; Fernández‐Ananín et al. [27]; Thomas et al. [28]
Costs			
Semaglutide monthly cost	$245 ($150, $649.42)		The White House [1], CMS [2], HHS [29]
Semaglutide price reduction (after patent expiration)	27% (20.25%, 33.75%)		FDA [30]
LSG initial surgery cost	$28,298.57 ($21,223.93, $35,373.21)		Alsumali et al. [31]
LSG major early complications cost	$53,159.74 ($39,869.80, $66,449.67)		Alsumali et al. [31]
LSG minor early complications cost	$1630.34 ($1222.75, $2037.92)		Alsumali et al. [31]
LSG major late complications cost	$58,530.60 ($43,897.95, $73,163.25)		Alsumali et al. [31]
LSG minor late complications cost	$1021.74 ($766.30, $1277.17)		Alsumali et al. [31]
Complications			
Semaglutide dropout rate	18.9% (17.01%, 20.79%)		Wilding et al. [24]
LSG major early complications rate	2.97% (0.81, 8.82%)	3.88% (1.07, 11.32%)	Alsumali et al. [31], Wood et al. [13]
LSG minor early complications rate	5.40% (0.96, 11.30%)	7.06% (1.27, 14.49%)	Alsumali et al. [31], Wood et al. [13]
LSG major late complications rate	0.09% (0.04, 0.41%)	0.12% (0.06, 0.53%)	Alsumali et al. [31], Wood et al. [13]
LSG minor late complications rate	0.39% (0.26, 1.01%)	0.51% (0.34, 1.29%)	Alsumali et al. [31], Wood et al. [13]

Abbreviation: LSG, laparoscopic sleeve gastrectomy.

### 2.3 Costs and Health Outcomes

The model incorporates healthcare costs associated with living in each health state, the monthly costs of semaglutide, the initial costs of LSG, and any costs resulting from surgical (major or minor) complications. Following the recommendation from the Second Panel on Cost‐Effectiveness in Health and Medicine, costs were converted to 2024 dollars using the overall personal healthcare cost expenditure deflator and personal consumption expenditures price index [32, 33]. Quality‐adjusted life‐years (QALYs) were derived from time spent in each health state, weighted by the quality‐of‐life estimate associated with each state. White and Black patients were assumed to have the same health state‐specific quality‐of‐life estimates. Patients who undergo surgery were assumed to have a quality‐of‐life reduction for 2 weeks post surgery and quality‐of‐life reductions for any major or minor complications associated with surgery (2 weeks each) [34]. We assumed there will be nonadherence to semaglutide based on intention‐to‐treat clinical trial data. We also assumed the most common side effects of semaglutide (nausea, vomiting, diarrhea) have negligible cost impacts but did account for their impact on quality of life in the first 4 months of the model [24, 35]. We discounted costs and QALYs at a monthly rate of 0.25% (annual rate of 3%). Although the Medicare GLP‐1 Bridge program is set to end on December 31, 2027, we assumed the lower semaglutide price will continue after that date. Since the model exceeds the patent expiration date of semaglutide in 2032, we assumed a price decrease after 66 months in the base‐case. We assumed the price drops by 27% when the patent expires but explored this decrease further in sensitivity analysis [30].

### 2.4 Conventional CEA

We estimated total costs and total QALYs for each intervention to calculate each incremental cost‐effectiveness ratio (ICER) for the conventional CEA. We assumed a willingness‐to‐pay (WTP) threshold of $100,000/QALY to choose the preferred strategy in the conventional CEA and to calculate the race‐subgroup‐specific net health benefit (NHB), which is required for DCEA calculations [36, 37].

### 2.5 Distributional CEA

To conduct a DCEA, we adopted an Atkinson‐based approach to represent the trade‐offs between efficiency and equity [38]. In this approach, for each intervention, unequally distributed outcomes in a population are translated into an “equally distributed equivalent” (EDE) value. Equation (1) shows how the EDE value is calculated, where *n* represents the total population size, *h*_i_ represents the average absolute NHB of each intervention for group *i*, and *ε* represents a decision‐maker's inequality aversion, where higher *ε* values indicate an increased preference for equity. The NHB converts total health outcomes and costs of an intervention into a single value measured in health units of QALYs according to the formula NHB = QALYs−Costs/WTP. The intervention with the highest EDE value is preferred. While there is no established inequality aversion parameter (*ε*) for the U.S., previous literature suggests a range of 0.5–3.0 [39, 40, 41, 42]. However, given more recent emerging data on population equity preferences, we also performed a sensitivity analysis on the ε value, using a threshold inequality aversion parameter (TIAP) approach to identify the *ε* value that would make an intervention distributionally cost‐effective [43, 44]. Following the precedent of previous DCEA literature, we primarily assumed the costs of each intervention are paid for proportionally by the White and Black populations in the model (proportional payment assumption). However, to capture the full distributional impact of each intervention, we also analyzed equity outcomes under a zero payment assumption (NHB = QALYs), in which the interventions are paid for by other taxpayers.

(1) $$\mathrm{EDE}={\left[\frac{1}{n}\sum_{i=1}^{n}{\left[h_{i}\right]}^{1-\varepsilon }\right]}^{\frac{1}{1-\varepsilon }}$$

### 2.6 Sensitivity Analysis

We performed deterministic one‐way sensitivity analysis, probabilistic sensitivity analysis (PSA), scenario analysis, and threshold analysis to understand how uncertainty in parameterization influences the results. One‐way sensitivity analysis estimates the sensitivity of model results to each input when pushed to high and low values. The PSA estimates the likelihood that each alternative is cost‐effective when accounting for uncertainty in all parameters simultaneously. Scenario analysis demonstrates model results under a particular fixed set of conditions (such as semaglutide price). Threshold analysis indicates the point at which a specific model input would need to change to make an alternative intervention conventionally cost‐effective or distributionally cost‐effective, and it includes the TIAP approach for the DCEA [44]. While threshold analysis is typically performed for the price of an intervention, we also conducted a threshold analysis on additional per‐person lifetime discounted QALYs associated with semaglutide, given recent findings demonstrating the positive impact of semaglutide on disease areas unrelated to obesity, such as substance use disorder [45].

## 3 Results

### 3.1 Conventional CEA

Base‐case results are displayed in Table 2. Semaglutide, compared to “do nothing,” has an ICER of approximately $7300/QALY. LSG, compared to “do nothing,” has an ICER of approximately $12,100/QALY. Compared to semaglutide, LSG produces a greater overall lifetime health benefit (per‐person QALYs of 9.08 versus 8.90) for a slightly higher lifetime cost (per‐person costs of $102,400 versus $92,400), and it is cost‐effective, with an ICER of $53,700/QALY.

**TABLE 2:** Base‐case results table for conventional CEA*.

Intervention	Population	25‐year discounted costs per person	25‐year discounted QALYs per person	ICER value (intervention vs. “do nothing”)	ICER value (LSG vs. semaglutide)
“Do nothing”	Overall	$ 80,655	7.29	—	—
	Black	$ 68,481	6.26	—	—
	White	$ 82,626	7.45	—	—
	Difference	$ 14,145	1.19	—	—
Semaglutide 2.4 mg	Overall	$ 92,389	8.90	$ 7290/QALY	—
	Black	$ 81,576	7.84	$ 8307/QALY	—
	White	$ 94,139	9.07	$ 7129/QALY	—
	Difference	$ 12,563	1.23	—	—
LSG	Overall	$ 102,402	9.08	$ 12,108/QALY	$ 53,675/QALY
	Black	$ 95,956	7.92	$ 16,629/QALY	$ 189,182/QALY
	White	$ 103,445	9.27	$ 11,443/QALY	$ 45,521/QALY
	Difference	$ 7489	1.35	—	—

Abbreviations: ICER, incremental cost‐effectiveness ratio; LSG, laparoscopic sleeve gastrectomy; QALY, quality‐adjusted life‐year.

* While we report race‐specific results and the difference between the Black and White populations here, we do not interpret them in the conventional cost‐effectiveness analysis because policy decisions must be applied across all racial subpopulations.

At the current WTP threshold, the price of semaglutide would need to be lowered by 44% to achieve cost‐effectiveness in comparison to LSG. We find that over a 25‐year period, an individual would need to gain an additional 0.15 discounted QALYs (from disease areas unrelated to obesity) for semaglutide to be considered cost‐effective compared to LSG.

One‐way sensitivity analysis results reveal heterogeneity in sensitivity to parameters based on the comparison groups, but the results were most consistently sensitive to the weight regain after LSG and the discount rate (Figures S1–S3). The PSA further underscores this variability in the results (cost‐effectiveness acceptability curve in Figure 3, scatterplot in Figure S4). At the WTP threshold of $100,000/QALY, LSG has a 57% probability and semaglutide has a 43% probability of being the most cost‐effective intervention.

**FIGURE 3:**
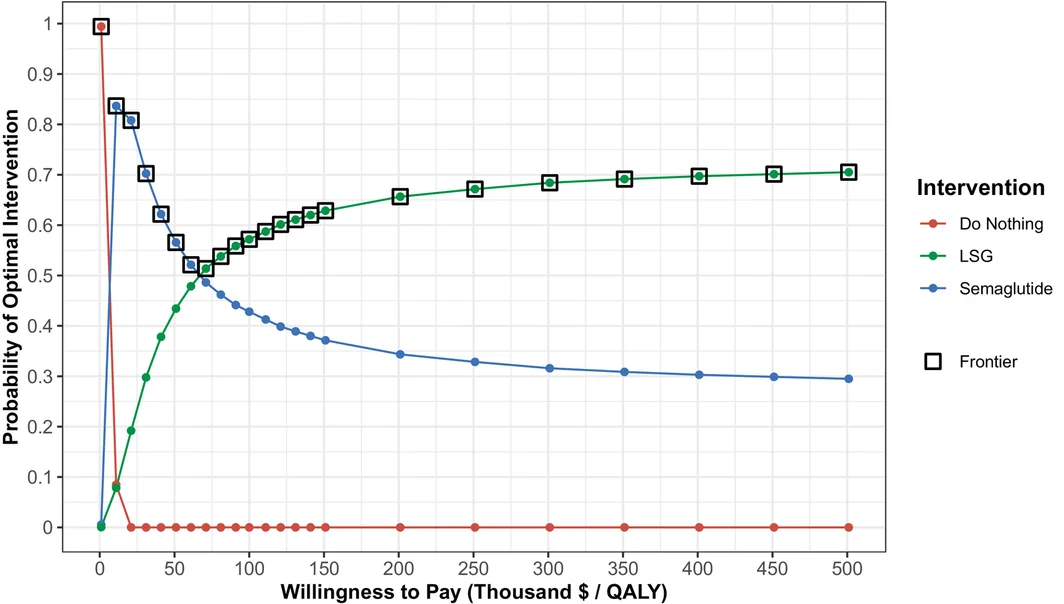
Cost‐effectiveness acceptability curve and cost‐effectiveness acceptability frontier for combined population. LSG, laparoscopic sleeve gastrectomy, QALY, quality‐adjusted life‐year. [Color figure can be viewed at wileyonlinelibrary.com]

Two scenario analyses were conducted on the price of semaglutide. The first scenario analysis represents an event in which the price of semaglutide remains at its previous net price ($649.42). Semaglutide becomes dominated by LSG, with higher costs and lower QALYs gained. A 79% reduction in price or an additional 0.71 discounted QALYs would be necessary for semaglutide to be considered cost‐effective over LSG from that higher price. At the previous price of semaglutide, LSG is the preferred intervention at nearly all WTP values, with a 95% probability of being the most cost‐effective intervention at the WTP threshold of $100,000/QALY (Figure S5). The second scenario analysis represents an event in which the price of semaglutide initially drops to its lowest negotiated value (for starter doses of the oral form), $150 per month. In this scenario, at the WTP threshold of $100,000/QALY, there is a 55% probability that semaglutide would be the most cost‐effective intervention, but as the WTP values increase, LSG becomes more likely to be the preferred intervention (Figure S6).

### 3.2 DCEA

Both Black and White populations would increase their QALYs with either intervention compared to the “do nothing” alternative. On average, each Black individual gains approximately 0.08 more QALYs and each White individual gains approximately 0.20 QALYs with LSG relative to semaglutide (Table 2). Scenario analysis indicates that the race‐specific model inputs that close the outcome gap the most are postoperative weight loss following LSG and late minor complications following LSG (Table S2).

Figures S7–S9 demonstrate the step‐by‐step calculations of the equity impact for each intervention under both the proportional payment and zero payment assumptions for the entire Black and White populations in the U.S., of which the study populations represent a subset. Both LSG and semaglutide interventions moderately increase the quality‐adjusted life expectancy (QALE) for the entire Black and White populations compared to the “do nothing” strategy (Figure S8). However, Figure S9 shows that semaglutide is marginally more equitable as an intervention under both payment assumptions.

Using the Atkinson social welfare index equation (Equation 1) and the proportional payment assumption, we find that the preferred decision at the current price is LSG over our assumed ε range of 0.5–3.0. Semaglutide's equity impact is outweighed by the economic efficiency of LSG, suggesting that semaglutide is not currently a distributionally cost‐effective intervention compared to LSG. With an *ε* value of 3.0, threshold analysis indicates that the initial price of semaglutide would need to be reduced by approximately 31% for semaglutide to be considered a distributionally cost‐effective intervention. With an *ε* value of 0.5, the price of semaglutide would need to be reduced by approximately 36% to reach distributional cost‐effectiveness (Figure S10). Alternatively, with an *ε* value of 0.5–3.0, the number of additional QALYs gained over a 25‐year period for all individuals (i.e., not race‐specific gains) would need to be approximately 0.09 discounted QALYs (*ε* = 3.0) to 0.11 discounted QALYs (*ε* = 0.5) to achieve distributional cost‐effectiveness (Figure S11). At its current price and effectiveness values, the TIAP (i.e., the *ε* value when semaglutide becomes the preferred intervention) is 14.72. Figure 4 displays the EDE values for both semaglutide and LSG interventions against increasing *ε* values.

**FIGURE 4:**
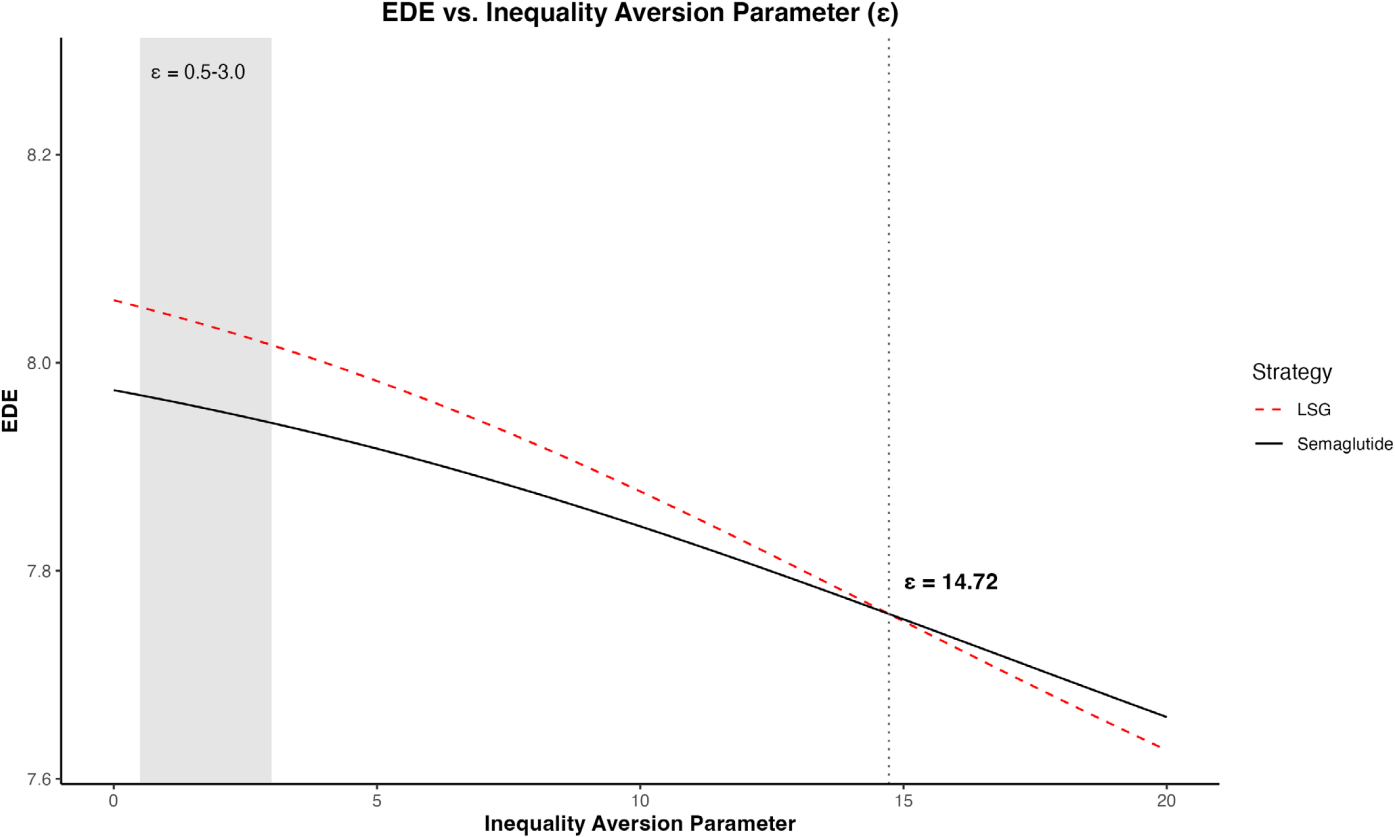
Equally distributed equivalent (EDE) values at different *ε* values. Decision rule is to choose intervention with highest EDE value. The EDE value weighs equity preferences along with conventional CEA parameters (costs, QALYs). The shaded area indicates our assumed *ε* range of 0.5–3.0. The gray dotted line indicates the threshold value of *ε* where semaglutide becomes the preferred intervention. CEA, cost‐effectiveness analysis, LSG, laparoscopic sleeve gastrectomy, QALY, quality‐adjusted life‐year. [Color figure can be viewed at wileyonlinelibrary.com]

## 4 Discussion

Our conventional cost‐effectiveness evaluation reveals that over a 25‐year time horizon, semaglutide, at its price of $245, is less expensive than LSG, but LSG is still conventionally cost‐effective compared to semaglutide. The uncertainty around this result depends on several factors, such as the price of semaglutide, ranging from LSG having a 57% probability of being conventionally cost‐effective (at semaglutide's negotiated price of $245) compared to 95% (using semaglutide's pre‐negotiation net price of $649.42). Either intervention is considered cost‐effective in comparison to the “do nothing” option. Additionally, since this analysis is from the healthcare perspective, it is likely that both semaglutide and LSG might be more cost‐effective if we consider only the patient perspective, since patients typically only bear a fraction of total healthcare costs. But this implication would depend on specific factors, such as the details of one's insurance coverage and individual‐level WTP for health outcomes, which population‐level policy decisions cannot be based on.

Regarding its distributional impact, semaglutide would reduce the Black‐White health disparity among this Medicare population compared to LSG. However, semaglutide's equity impact is not large enough to offset its worse conventional cost‐effectiveness outcomes in the overall population relative to LSG. Using an Atkinson‐based approach, we find that semaglutide is not a distributionally cost‐effective intervention in our assumed *ε* range of 0.5–3.0 compared to LSG. Using a TIAP approach, we find that semaglutide would be distributionally cost‐effective if the decision‐maker's inequality aversion value, *ε*, is at least 14.72. Although well outside our assumed ε range, a recent survey of the general U.S. population finds a higher median *ε* value of 12 with a wide interquartile range, so depending on the decision‐maker's inequality aversion preferences, semaglutide could be distributionally cost‐effective in comparison to LSG [43].

Threshold analyses reveal that changes to the price or additional QALY gains of semaglutide may render it cost‐effective or distributionally cost‐effective compared to LSG. The initial price of semaglutide could be lowered by 44% for it to achieve an ICER value at the WTP threshold ($100,000/QALY), and to achieve distributional cost‐effectiveness with a range of ε values from 0.5 to 3.0, the initial price could be lowered by 31%–36%. By conventional standards, individuals would need to gain 0.15 more discounted lifetime QALYs for semaglutide to be considered cost‐effective. To achieve distributional cost‐effectiveness, each individual in the model would need to gain an additional 0.09–0.11 discounted QALYs (ranges correspond to ε values from 0.5 to 3.0). For many high‐risk individuals with health conditions unrelated to obesity, this is likely to be a plausible amount of additional health benefit from semaglutide. It is beyond the scope of this analysis to study this question further; however, this may be an area to consider for future research.

Our study has several limitations. First, we made key assumptions to build and implement this model. Data on the Black population in the literature are sparse, and most model parameters for the Black population had to be calculated using several different data sources and may be subject to error. Data on Medicare beneficiaries with obesity are limited as well—most studies report outcomes for a general adult population, which is why we adjusted these parameters for an older adult population. Additionally, because we adjusted data to fit our study population, our results are not generalizable for a broader population, which limits the applicability of our results. Future research could repeat our study for a larger study population, particularly as eligibility criteria for GLP‐1 agonists and bariatric surgery change. Second, because our study population has an obesity‐related cardiovascular condition, we had to adjust mortality rates to reflect this disease history. To obtain estimates for a Black and older population with prior cardiovascular disease, we had to make assumptions of independence that may not fully hold. Details on parameter estimations and calculations can be found in the online Supporting Information. Third, to adequately compare treatment options to one another, we assumed patients would have full access to either semaglutide or LSG, which may not be true in the real world. Fourth, most studies reporting semaglutide or LSG outcomes have a relatively short time horizon, which required the extrapolation of certain parameters over a longer time period. Fifth, even with the new price reductions in the cost of semaglutide, we recognize that there is still considerable uncertainty in the price of semaglutide moving forward, especially when it loses its patent protection. While we attempted to model a wide‐ranging price through sensitivity analysis and adhered to historical price reduction patterns, it is unclear how federal drug price negotiations or market conditions may change in the future. Finally, DCEA methodology requires the division of the study population into subgroups, which may also lead to some limitations in analysis. Our choice to classify subgroups by race is due to both policy relevance and data availability; however, there are other distinct, but correlated, socioeconomic factors (such as income or education) or systemic issues that could drive distributional differences in obesity outcomes. As more data become available, future DCEA studies should look to incorporate other socioeconomic groupings that better relate to the underlying causes of health disparities rather than solely race as a proxy variable.

Despite limitations, we believe our study is highly unique in its timely evaluation of both the economic and distributional impact of a semaglutide intervention under its new price for the two older racial subgroups with the highest levels of obesity. We compare semaglutide to a clinically relevant alternative intervention for obesity (LSG), as well as a highly realistic obesity intervention (doing nothing). Our conclusions for the overall study population demonstrate the efficiency of both LSG and semaglutide under its new price, and we highlight important subgroup and equity impacts of semaglutide that may be relevant for decision‐makers to consider.

## 5 Conclusion

Our model‐based analyses suggest that both semaglutide and LSG are conventionally cost‐effective interventions for obesity compared to a “do nothing” option. This suggests both treatments should be available to Medicare patients with at least Class II obesity and cardiovascular disease. Results suggest that LSG is more likely to be cost‐effective, particularly at higher (pre‐negotiation) semaglutide prices. Furthermore, while we find that semaglutide is a more equitable intervention than LSG, its marginal equity gains are outweighed by its conventional cost‐effectiveness outcomes. Thus, semaglutide is not distributionally cost‐effective compared to LSG under our assumed inequality aversion, although these results may depend on how decision‐makers weigh equity impacts against conventional cost‐effectiveness. Further price reductions or additional QALY gains would be necessary to make semaglutide the preferred intervention over LSG using either a conventional or distributional CEA approach.

## Author Contributions

N.S.K. had full access to all the data in the study and takes responsibility for the integrity of the data and the accuracy of the data analysis. The authors of this article contributed to the study in the following manner: Concept and design: N.S.K., A.P. Acquisition, analysis, or interpretation of the data: N.S.K., A.P., D.H., C.R., M.G. Drafting of the manuscript: All authors. Data visualization: N.S.K. Critical revision of the manuscript for important intellectual content: All authors. Supervision: A.P., D.H., J.S.M. All authors approved the final version of the manuscript.

## Funding

Dr. Jeffery S. McCullough is the recipient of AWD025741 from the National Institute for Health Care Reform. The funding source had no role in the study design; collection, analysis, or interpretation of the data; writing of the manuscript; or the decision to submit the manuscript for publication.

## Conflicts of Interest

The authors declare no conflicts of interest.

## Supporting information


**Table S1:** Full parameter list.
**Table S2:** Scenario analysis results.
**Figures S1–S3:** One‐way sensitivity analysis results.
**Figure S4:** Probabilistic sensitivity analysis results for Black and White populations.
**Figure S5:** Cost‐effectiveness acceptability curve results with previous price of semaglutide (net price = $649.42).
**Figure S6:** Cost‐effectiveness acceptability curve results with lowest price of semaglutide ($150).
**Figures S7–S9:** Equity results and calculations.
**Figure S10:** Heatmap of semaglutide price against increasing inequality aversion parameters (ε).
**Figure S11:** Heatmap of additional lifetime QALYs against increasing inequality aversion parameters (ε).

## Data Availability

Statistical code and dataset: Restricted access is available through written agreements with the corresponding author (e‐mail neetik@umich.edu).

## References

[1] The White House , “Fact Sheet: President Donald J. Trump Announces Major Developments in Bringing Most‐Favored‐Nation Pricing to American Patients,” published November 6, (2025), https://www.whitehouse.gov/fact‐sheets/2025/11/fact‐sheet‐president‐donald‐j‐trump‐announces‐major‐developments‐in‐bringing‐most‐favored‐nation‐pricing‐to‐american‐patients/.

[2] U.S. Department of Health and Human Services, Centers for Medicare & Medicaid Services , “Medicare GLP‐1 Bridge: Expectations and Frequently Asked Questions for Part D Sponsors,” published June 10, (2026), https://www.cms.gov/files/document/medicare‐glp‐1‐bridge‐expectations‐faqs.pdf.

[3] S. J. Atlas , K. Kim , M. Beinfeld , et al., Institute for Clinical and Economic Review , “Medications for Obesity Management: Effectiveness and Value; Final Evidence Report,” published October 20, (2022), last modified December 22, 2023, https://icer.org/assessment/obesity‐management‐2022.

[4] M. Haseeb , J. Chhatwal , J. Xiao , P. Jirapinyo , and C. C. Thompson , “Semaglutide vs. Endoscopic Sleeve Gastroplasty for Weight Loss,” JAMA Network Open 7, no. 4 (2024): e246221, 10.1001/jamanetworkopen.2024.6221. PMC11015347 38607627

[5] J. H. Hwang , N. Laiteerapong , E. S. Huang , and D. D. Kim , “Lifetime Health Effects and Cost‐Effectiveness of Tirzepatide and Semaglutide in US Adults,” JAMA Health Forum 6, no. 3 (2025): e245586, 10.1001/jamahealthforum.2024.5586. PMC11909610 40085108

[6] G. Lin , W. Lee , S. Fahim , et al., “Semaglutide and Tirzepatide for Obesity: Effectiveness and Value; Draft Evidence Report,”.

[7] Centers for Disease Control and Prevention , “About Obesity,” last modified January 23, 2024, https://www.cdc.gov/obesity/php/about/index.html.

[8] S. D. Emmerich , C. D. Fryar , B. Stierman , and C. L. Ogden , Obesity and Severe Obesity Prevalence in Adults: United States, August 2021‐August 2023 , NCHS Data Brief, no. 508 (National Center for Health Statistics, 2024), 10.15620/cdc/159281. PMC11744423 39808758

[9] Centers for Medicare and Medicaid Services, Office of Minority Health , “Obesity Disparities in Medicare Fee‐For‐Service Beneficiaries [Data Snapshot],” published January (2022), https://www.cms.gov/files/document/omh‐datasnapshot‐obesity.pdf.

[10] K. Seeras , S. Sankararaman , and P. P. Lopez , “Sleeve Gastrectomy,” In StatPearls (StatPearls Publishing, 2023), https://www.ncbi.nlm.nih.gov/books/NBK519035/. 30085577

[11] M. K. Masanam , D. A. Grossman , J. Neary , and Y. R. Alimi , “Disparities in the Impact of Access to and Outcomes of Bariatric Surgery Among Different Ethnoracial and Socioeconomic Populations: A Narrative Review of the Literature,” Annals of Laparoscopic and Endoscopic Surgery 8 (2023): 34, https://ales.amegroups.org/article/view/9464.

[12] P. Wisniowski and K. Samakar , “Racial Disparities in the Outcomes of Bariatric Surgery,” Current Surgery Reports 11, no. 10 (2023): 270–276, 10.1007/s40137-023-00372-x.

[13] M. H. Wood , A. M. Carlin , A. A. Ghaferi , et al., “Association of Race With Bariatric Surgery Outcomes,” JAMA Surgery 154, no. 5 (2019): e190029, 10.1001/jamasurg.2019.0029. PMC6537777 30840063

[14] J. Cubanski , T. Neuman , N. Sroczynski , and A. Damico , “A New Use for Wegovy Opens the Door to Medicare Coverage for Millions of People With Obesity,” KFF, published April 24, (2024), https://www.kff.org/medicare/issue‐brief/a‐new‐use‐for‐wegovy‐opens‐the‐door‐to‐medicare‐coverage‐for‐millions‐of‐people‐with‐obesity/.

[15] Food and Drug Administration , “Drug Trial Snapshot: Ozempic,” accessed July 17. 2025, https://web.archive.org/web/20250212044919/http://fda.gov/drugs/drug‐approvals‐and‐databases/drug‐trial‐snapshot‐ozempic.

[16] M. Sculpher , “Subgroups and Heterogeneity in Cost‐Effectiveness Analysis,” PharmacoEconomics 26, no. 9 (2008): 799–806, 10.2165/00019053-200826090-00009. 18767899

[17] M. Asaria , S. Griffin , and R. Cookson , “Distributional Cost‐Effectiveness Analysis: A Tutorial,” Medical Decision Making 36, no. 1 (2016): 8–19, 10.1177/0272989X15583266. PMC4853814 25908564

[18] T. B. Washington , V. R. Johnson , K. Kendrick , et al., “Disparities in Access and Quality of Obesity Care,” Gastroenterology Clinics of North America 52, no. 2 (2023): 429–441, 10.1016/j.gtc.2023.02.003. PMC10198472 37197884

[19] A. Alsuhibani , J. R. Thompson , P. R. Wigle , et al., “Metabolic and Bariatric Surgery Utilization Trends in the United States: Evidence From 2012 to 2021 National Electronic Medical Records Network,” Annals of Surgery Open 4, no. 4 (2023): e317, 10.1097/AS9.0000000000000317. PMC10735086 38144499

[20] Centers for Medicare and Medicaid Services ,“Bariatric Surgery for the Treatment of Morbid Obesity—Facility Certification Requirement,” Report CAG‐00250R3, published September 24, (2013), https://www.cms.gov/medicare‐coverage‐database/view/ncacal‐decision‐memo.aspx?proposed=N&NCAId=266.

[21] Y. Tang , Z. Zhang , and X. Liu , “Lifestyle Modifications and Control of Cardiovascular Risk Factors in Older Adults With Hypertension: From NHANES 1999‐2018,” BMC Geriatrics 25, no. 1 (2025): 537, 10.1186/s12877-025-06204-0. PMC12272962 40681986

[22] D. Husereau , M. Drummond , F. Augustovski , et al., “Consolidated Health Economic Evaluation Reporting Standards (CHEERS) 2022 Explanation and Elaboration: A Report of the ISPOR CHEERS II Good Practices Task Force,” Value in Health 25, no. 1 (2022): 10–31, 10.1016/j.jval.2021.10.008. 35031088

[23] A. Zajacova , S. Huzurbazar , M. Greenwood , and H. Nguyen , “Long‐Term BMI Trajectories and Health in Older Adults: Hierarchical Clustering of Functional Curves,” Journal of Aging and Health 27, no. 8 (2015): 1443–1461, 10.1177/0898264315584329. PMC5436799 25953813

[24] J. P. H. Wilding , R. L. Batterham , S. Calanna , et al., “Once‐Weekly Semaglutide in Adults With Overweight or Obesity,” New England Journal of Medicine 384, no. 11 (2021): 989–1002, 10.1056/NEJMoa2032183. 33567185

[25] D. H. Ryan , I. Lingvay , J. Deanfield , et al., “Long‐Term Weight Loss Effects of Semaglutide in Obesity Without Diabetes in the SELECT Trial,” Nature Medicine 30, no. 7 (2024): 2049–2057, 10.1038/s41591-024-02996-7. PMC11271387 38740993

[26] P. Salminen , S. Grönroos , M. Helmiö , et al., “Effect of Laparoscopic Sleeve Gastrectomy vs Roux‐En‐Y Gastric Bypass on Weight Loss, Comorbidities, and Reflux at 10 Years in Adult Patients With Obesity: The SLEEVEPASS Randomized Clinical Trial,” JAMA Surgery 157, no. 8 (2022): 656–666, 10.1001/jamasurg.2022.2229. PMC9218929 35731535

[27] S. Fernández‐Ananín , E. Ballester , B. Gonzalo , et al., “Is Sleeve Gastrectomy as Effective in Older Patients as in Younger Patients? A Comparative Analysis of Weight Loss, Related Comorbidities, and Medication Requirements,” Obesity Surgery 32, no. 6 (2022): 1909–1917, 10.1007/s11695-022-05940-1. PMC9072441 35411452

[28] D. D. Thomas , W. A. Anderson , C. M. Apovian , et al., “Weight Recidivism After Roux‐En‐Y Gastric Bypass Surgery: An 11‐Year Experience in a Multiethnic Medical Center,” Obesity (Silver Spring) 27, no. 2 (2019): 217–225, 10.1002/oby.22360. PMC6345597 30421862

[29] Assistant Secretary for Planning and Evaluation: Office of Health Policy , Medicare Coverage of Anti‐Obesity Medications, Issue Brief no. HP‐2024‐25 (U.S. Department of Health and Human Services, 2024), https://aspe.hhs.gov/sites/default/files/documents/127bd5b3347b34be31ac5c6b5ed30e6a/medicare‐coverage‐anti‐obesity‐meds.pdf.

[30] R. Conrad and R. Lutter , Generic Competition and Drug Prices: New Evidence Linking Greater Generic Competition and Lower Generic Drug Prices (Food and Drug Administration, 2019), https://www.fda.gov/media/133509/download.

[31] A. Alsumali , T. Eguale , S. Bairdain , and M. Samnaliev , “Cost‐Effectiveness Analysis of Bariatric Surgery for Morbid Obesity,” Obesity Surgery 28, no. 8 (2018): 2203–2214, 10.1007/s11695-017-3100-0. 29335933

[32] A. Basu , “Estimating Costs and Valuations of Non‐Health Benefits in Cost‐Effectiveness Analysis,” in Cost‐Effectiveness in Health and Medicine, ed. P. J. Neumann , T. G. Ganiats , L. B. Russell , G. D. Sanders , and J. E. Siegel (Oxford University Press, 2016), 201–236, 10.1093/acprof:oso/9780190492939.003.0008.

[33] Agency for Healthcare Research and Quality , “Using Appropriate Price Indices for Analyses of Health Care Expenditures or Income Across Multiple Years,” Medical Expenditure Panel Survey (MEPS) website, https://meps.ahrq.gov/about_meps/Price_Index.shtml.

[34] J. Campbell , L. A. McGarry , S. A. Shikora , B. C. Hale , J. T. Lee , and M. C. Weinstein , “Cost‐Effectiveness of Laparoscopic Gastric Banding and Bypass for Morbid Obesity,” American Journal of Managed Care 16, no. 7 (2010): e174–e187. 20645663

[35] M. F. Pollack , F. W. Purayidathil , S. C. Bolge , and S. A. Williams , “Patient‐Reported Tolerability Issues With Oral Antidiabetic Agents: Associations With Adherence; Treatment Satisfaction and Health‐Related Quality of Life,” Diabetes Research and Clinical Practice 87, no. 2 (2010): 204–210, 10.1016/j.diabres.2009.11.023. 20036433

[36] P. J. Neumann and D. D. Kim , “Cost‐Effectiveness Thresholds Used by Study Authors, 1990‐2021,” JAMA 329, no. 15 (2023): 1312–1314, 10.1001/jama.2023.1792. PMC10114019 37071104

[37] D. J. Vanness , J. Lomas , and H. Ahn , “A Health Opportunity Cost Threshold for Cost‐Effectiveness Analysis in the United States,” Annals of Internal Medicine 174, no. 1 (2021): 25–32, 10.7326/M20-1392. 33136426

[38] A. B. Atkinson , “On the Measurement of Inequality,” Journal of Economic Theory 2, no. 3 (1970): 244–263, 10.1016/0022-0531(70)90039-6.

[39] I. Kawachi and B. P. Kennedy , “The Relationship of Income Inequality to Mortality: Does the Choice of Indicator Matter?,” Social Science and Medicine 45, no. 7 (1997): 1121–1127, 10.1016/S0277-9536(97)00044-0. 9257403

[40] M. Cropper , A. Krupnick , and W. Raich , “Preferences for Equality in Environmental Outcomes,” Resources for the Future Discussion Paper 16‐36, published September 2, (2016), https://papers.ssrn.com/sol3/papers.cfm?abstract_id=2851945.

[41] B. Glassman , “Multidimensional Inequality: Measurement and Analysis Using the American Community Survey,” U.S. Census Bureau, Working Paper No. SEHSD‐2019–17, published March 2019, https://www.census.gov/library/working‐papers/2019/demo/SEHSD‐WP2019‐17.html.

[42] J. I. Levy , S. L. Greco , S. J. Melly , and N. Mukhi , “Evaluating Efficiency‐Equality Tradeoffs for Mobile Source Control Strategies in an Urban Area,” Risk Analysis 29, no. 1 (2009): 34–47. 10.1111/j.1539-6924.2008.01119.x PMC3690594 18793281

[43] J. F. Slejko , S. Ricci , R. Cookson , and S. Kowal , “Health Inequality Aversion in the United States,” Value in Health 29 (2025): 129–138. 10.1016/j.jval.2025.08.015 40939702

[44] A. Pandya , J. Zhu , A. Luviano , L. P. James , and G. Goshua , “A Threshold Inequality Aversion Parameter Approach to Interpret Distributional Cost‐Effectiveness Analysis Results,” Value in Health 9, no. 6 (2025): 946–952, 10.1016/j.jval.2025.09.3064. PMC12684637 41093070

[45] Y. Xie , T. Choi , and Z. Al‐Aly , “Mapping the Effectiveness and Risks of GLP‐1 Receptor Agonists,” Nature Medicine 31, no. 3 (2025): 951–962, 10.1038/s41591-024-03412-w. 39833406

