# Supplementary material for: The Conventional Cost‐Effectiveness and Distributional Cost‐Effectiveness of Semaglutide for Obesity Treatment
Source: Obesity (Silver Spring). 2026 Oct 7;34(11):2146–55. doi: 10.1002/oby.70311 (PMC13716183; doi:10.1002/oby.70311)
Supplement: Supplementary file 1 [file OBY-34-2146-s001.docx]

**Title: The Conventional Cost-Effectiveness and Distributional Cost-Effectiveness of Semaglutide for Obesity Treatment**

**Running Title: An Equity-Based Economic Evaluation of Semaglutide**

Neeti S. Kulkarni^1^, David Hutton^1^, Corwin Rhyan^1^, Jeffrey S. McCullough^1^, Michaela Greenlee^2^, Ankur Pandya^3^

^1^ Department of Health Management and Policy, University of Michigan School of Public Health, Ann Arbor, MI.

^2^ Department of Global Health and Population, Harvard T.H. Chan School of Public Health, Boston, MA.

^3^ Department of Health Policy and Management, Harvard T.H. Chan School of Public Health, Boston, MA.

**Financial Support Information:** Dr. Jeffery S. McCullough is the recipient of AWD025741 from the National Institute for Health Care Reform.

**Corresponding Author:** Neeti Kulkarni, BA, Department of Health Management and Policy, University of Michigan School of Public Health, 1415 Washington Heights, Ann Arbor, MI 48109HeHe; neetik@umich.edu.

**Supplementary Material**

**Contents**

Methods……………………………………………………………………………………………3

Parameter assumptions and calculations…………………………………………………..3

Supplementary Tables and Figures………………………………………………………………12

Table S1: Full Parameter List……………………………………………………………12

Figures S1-S3: One-way sensitivity analysis results…………………………………….18

Figure S4: PSA results for Black and White populations………………………………..21

Figure S5: CEAC Results with Previous Price of Semaglutide………………………….21

Figure S6: CEAC Results with Lowest Price of Semaglutide…………………………...22

Figures S7-S9: Equity calculations and results…………………………………………..22

Figure S10: Heatmap of semaglutide price………………………………………………24

Figure S11: Heatmap of additional lifetime QALYs…………………………………….24

Table S2: Scenario analysis results ……………………………………………………...25

References………………………………………………………………………………………..26

**Parameter Assumptions and Calculations**

***Deriving the Starting Populations***

Under the Medicare indication for Wegovy® (semaglutide) passed in April 2024 for individuals who are overweight or obese with an established cardiovascular condition, Cubanski et al. (2024) estimates that 3.6 million individuals would be eligible for Wegovy®, and of that 3.6 million, 1.7 million individuals do not have Type 2 diabetes (1). When we break down this estimate by the racial distribution of Medicare beneficiaries (as reported by KFF), we have approximately 1,237,600 White individuals and 170,000 Black individuals who would be eligible for semaglutide under the new indication (2). According to KFF, 49% of Medicare beneficiaries are aged 65-74 years (3). If we assume an equal distribution of the population at each age in this group, 29.4% of the population is aged 65-70. Therefore, approximately 363,854 White individuals and 49,980 Black individuals are eligible for semaglutide under Medicare’s new indication. To also be eligible for LSG, which defines the population of interest for our simulation model, individuals must be at least Obesity Class II (BMI ≥ 35). Suehs et al. (2017) report the distribution of Medicare beneficiaries in each obesity class by race (4). Suehs et al. (2017) do not divide Class III obesity into different groups, so we use the distributions given in Campbell et al. (2010) only for Class III.I and III.II (5). For White individuals, 11.91% are Obesity Class II, 9.97% are Obesity Class III.I and 1.66% are Obesity Class III.II. For Black individuals, 13.86% are Obesity Class II, 11.90% are Obesity Class III.I, and 1.98% are Obesity Class III.II. Note that these distributions do not sum to 100% because we also have overweight and Obese Class I included in the distribution calculation. Ultimately, this gives the following starting distributions:

White:

| **White Obesity Classes** | **Population Counts** |
| --- | --- |
| Obesity Class II | 43,353 |
| Obesity Class III.I | 36,277 |
| Obesity Class III.II | 6,046 |

Black:

| **Black Obesity Classes** | **Population Counts** |
| --- | --- |
| Obesity Class II | 6,927 |
| Obesity Class III.I | 5,947 |
| Obesity Class III.II | 991 |

***Calculating Weight Trajectories***

‘Do Nothing’

*White calculation*

Using data from [Zajacova et al. (2017](https://pmc.ncbi.nlm.nih.gov/articles/PMC5436799/)), a longitudinal study of BMI trajectories in older adults, we consider individuals in the gaining category (who start overweight and gradually gain weight over 18 years to go into the obese category) to be most closely aligned with our study population (6). Therefore, we calculate monthly BMI change to be (32.0 (BMI in 2010) – 26.8 (BMI in 1992))/18/12 = 0.024.

*Black calculation*

We assume the Black population gains the same amount of weight in the ‘do nothing’ intervention as the White population.

Semaglutide

Note: Based on a recent meta-analysis published by Alexander et al. (2026), we adjust semaglutide efficacy by age but not by race (7). While the meta-analysis finds no heterogeneity across age subgroups, it only evaluates studies for patients with Type 2 diabetes, and more than half of the studies look at other GLP-1 agonists than semaglutide (such as liraglutide). Meanwhile, the studies included for the race analysis are mostly semaglutide studies for a non-diabetic population, which fits our study population more closely. After consulting with a clinical expert, it was determined that we should adjust semaglutide efficacy for age but not for race.

*White calculation*

Wilding et al. (2021) find that over a 68-week clinical trial, participants receiving only 2.4 mg of semaglutide lost -4.61 BMI points (8). We apply this linearly to be (-4.61/68)*4.35(number of weeks in a month) = -0.29 BMI points lost/month in year 1 of the model. However, Wilding et al. (2021) primarily studies adults at a younger age than our study population. To adjust for age-effects, a multiplier of 0.872 is applied to the estimate of -0.29 to obtain -0.26. The multiplier comes from Ryan et al. (2024) where the estimated treatment difference between the 65 to <75 age group and the <55 age group and is -8.10/-9.29 = 0.872 (9). The reason the estimates from Ryan et al. (2024) are not used for the primary estimate is because change in BMI is not reported in this trial, which we need to define the states of the model.

Based on clinical opinion, we assume that when adhering to semaglutide, no weight regain will occur.

*Black population*

As stated above, we do not adjust semaglutide efficacy for the Black population.

We also assume the Black population will not regain any weight when adhering to semaglutide.

LSG surgery

*White calculation*

Salminen et al. (2022) give BMI trajectories from the SLEEVEPASS RCT up to 10 years post-operation (10). From baseline to month 6, the mean BMI decreased from 47.3 to 35.8, which we apply linearly to be (35.8-47.3)/12 = -1.92 BMI points /month. From month 6 to month 12 (year 1), the mean BMI decreased from 35.8 to 34.4, which we apply to be (34.4-35.8)/12 = -0.23 BMI points /month. From year 1 to year 5, BMI increased from 34.4 to 36.5, which we apply as (36.5-34.4)/48 = 0.04375 BMI points/month. Because the mean age of this RCT is 48 years, we use data from Fernandez-Ananin et al. (2022) to adjust the BMI trajectories for an older-age population (11). They find that the older population lost approximately -0.99 BMI points/month vs. the younger population lost approximately -1.17 BMI points/month. This gives a multiplier of -0.99/-1.17 = 0.846 to adjust for an older-age population. For years 2-5, the older and younger age populations gained the same amount of BMI points, so no adjustment is needed. Since Fernandez-Ananin et al. (2022) only report 5-year results, we assume that younger and older age populations have the same rate of weight regain after 5 years as well.

Thus, the amount of BMI points lost the first 6 months =

-1.92*0.846 = -1.62 BMI points/month.

The amount of BMI points lost in the second 6 months = -0.23*0.846 = -0.20 BMI points/month.

The rate of weight regain in Y2-Y5 is 0.044.

Salminen et al. (2022) report a rate of weight regain of (37.8-36.5)/12 = 0.022 in Y6-10. We assume that this rate of weight regain continues until the end of the model (Y25 or death, whichever comes first).

*Black calculation*

Wood et al. (2019) find that 1 year following bariatric surgery, Black patients had a lower mean total body weight loss compared to White patients (32.0 kg vs. 38.3 kg), so a multiplier of 32.0/38.3 = 0.84 is applied to the BMI change estimate for the White population:

0.84*-1.62 = -1.36 BMI points/month for the first 6 months

and 0.84*-0.20 = -0.17 BMI points/month for the second 6 months (12).

We assume the Black population has the same rate of postoperative weight regain as the White population.

***Converting BMI point changes to health state transitions***

Individuals are assumed to start at the median BMI for each health state in the starting population (e.g.: BMI = 37.5 for Obesity Class II and BMI = 45 for Obesity Class III.I). This implies that for an individual to move from the median Class II BMI to the Class III.I boundary, they would need to gain 2.5 BMI points. For an individual to move from the median Class III.I BMI to the Class II boundry, they would need lose 5 BMI points. We assume individuals change BMI points at a constant monthly rate. For example, individuals lose 0.26 BMI points per month with semaglutide. We convert this to a rate: for an individual moving from Class III.I to Class II, their rate would be 1/(5/0.26) = 0.052. We then convert this to a probability = 1-exp(-0.052) = 0.0507.

***Calculating Complications, Side Effects, and Dropout***

Semaglutide

*White calculation*

Given that we use intention-to-treat (ITT) data from Wilding et al. (2021), we assume that the effectiveness (and subsequent quality-of-life utility estimates) then implicitly account for the dropout rate and any potential weight regain. For reference, 81.1% adhered (18.9% non-adherence rate) to treatment in Wilding et al. (2021)—a large percentage of those who did not complete treatment withdrew due to adverse events (8).

Based on reported side effects from Wilding et al. (2021), we assume that 44.2% of the study population will experience minor side effects of semaglutide such as nausea, vomiting, or diarrhea. Pollack et al. (2010) report the same quality-of-life impact from each side effect (-0.02 utility decrement) (13). We assume everyone who experiences diarrhea and vomiting will also experience nausea (therefore we use the estimated proportion of the population that experienced nausea in Wilding et al. (2021)). Wilding et al. (2021) report that most side effects are experienced during the dose escalation period (the first 16 weeks) and subsided with time. Thus, we assume that patients experience side effects for only the first 4 months of the model.

*Black calculation*

We assume the Black population has the same dropout rate and side effects rate as the White population. While the same dropout rate may not be reflective of real-world circumstances, our model assumes an idealistic scenario of perfect access to semaglutide for all racial subgroups, and therefore the same rate of dropout for reasons only related to major complications.

LSG

*White calculation*

Complications are sorted into 4 categories following the precedence of Alsumali et al. (2018): early minor, early major, late minor, late major (14). Early complications could occur only within the first month of surgery; thereafter all complications are considered late. The rate of early minor complications is 5.40% and the rate of early major complications is 2.97%. The rate of late minor complications is 4.70% and the rate of late major complications is 1.10%. While this estimate is for a population aged 18-74, an older population must have a reasonably good level of health to qualify for bariatric surgery, so we do not adjust the complication rates any further for an older population.

While some models (including Alsumali et al. (2018)) include a minimal 30-day surgical-related mortality (<1%), we assume no mortality related to surgery. Both studies we use to calculate effectiveness of surgery, Salminen et al. (2022) and Fernandez-Ananin et al. (2022), do not have any surgical-related deaths in their studies, and expert opinion has confirmed that any post-operative death is likely to be already accounted for through the all-cause mortality rates in the model (10,11).

*Black calculation*

The complications for the Black population are further adjusted by OR for any complication (1.33) and for serious complications (1.32) using estimates from Wood et al. (2019) (12). Thus, the odds calculated above are adjusted with the ORs for the Black population.

Thus odds_early minor Black_ = (odds_early minor White_ *OR_minor_) = (0.0540/(1-0.0540) *1.33)=0.076

probability_early minor Black_ = (odds_early minor Black_/(1+odds_early minor Black_))= 0.076 /(1+0.076) = 7.06%

odds_early major Black_ = (odds_early major White_ *OR_major_)= 0.0297/(1-0.0297)=0.031*1.32

probability_early major Black_ =(odds_early major Black_/(1+odds_early major Black_))= 0.041/(1+0.041) =3.88%

odds_late minor Black_ = (odds_late minor White_ *OR_minor_) =0.0470/(1-0.0470)=0.049*1.33

probability_late minor Black_ = (odds_late minor Black_/(1+odds_late minor Black_))= 0.065/(1+0.065) =6.16% =

odds_late major Black_ = (odds_late major White_ *OR_major_)= 0.0110/(1-0.0110)=0.011*1.32

probability_late major Black_ =(odds_late major Black_/(1+odds_late major Black_))= 0.015/(1+0.015) =1.45%

The probability of early minor complications for the Black population then becomes 7.06%, the probability of early major complications becomes 3.88%, the probability of late minor complications becomes 6.16%, and the probability of late major complications becomes 1.45%.

***Obtaining Long-Term Mortality Rates***

*White calculation*

Our study population starts in Obesity Class II or higher with an established cardiovascular condition. Therefore, we need to calculate the mortality rate for different obesity classes with established cardiovascular risk. We obtain mortality estimates by BMI group for healthy White adults in age groups 60-69 years and 70-84 from Berrington de Gonzalez et al. (2011) (15). Although individuals in the model may live until they are 90-95 years old (if they do not die before the end of the model), we assume the mortality rates for the 70–84 age group would apply after the age of 84 as well (since Berrington de Gonzalez et al. (2011) do not report any older age groups). In some cases, Berrington de Gonzalez et al. (2011) report mortality rates for two BMI groups that fall within one obesity class, so the median mortality rate is used between the two groups. They are then adjusted with a multiplier for a cardiovascular condition from Pandya et al. (2017) (16). Pandya et al. (2017) report 3 different multipliers—post-stroke all-cause mortality multiplier (2.3), post-coronary heart disease (CHD) all-cause mortality multiplier (male) (1.6), and post-CHD all-cause mortality multiplier (female) (2.1). According to KFF, 55% of Medicare beneficiaries are women and 45% are men (17). First, a weighted average of post-CHD all-cause mortality is found to be 0.55(2.1) + 0.45(1.6) = 1.875. Then an average of the two ratios is taken to be 2.3 + 1.875 / 2 = 2.09, which is then used as the multiplier for an “established CVD condition.” Therefore, mortality rates for the White healthy population are multiplied by 2.09 to obtain for the age group 60-69 years:

| **Obesity group** | **Mortality rate for healthy White adults** | **Mortality rate for CVD-risk White adults** |
| --- | --- | --- |
| Non-obese | 0.82% | 1.71% |
| Overweight | 0.89% | 1.86% |
| Obesity Class I | 1.10% | 2.30% |
| Obesity Class II | 1.45% | 3.03% |
| Obesity Class III.I | 1.86% | 3.89% |
| Obesity Class III.II | 2.27% | 4.74% |

and for the age group 70-84 years:

| **Obesity group** | **Mortality rate for healthy White adults** | **Mortality rate for CVD-risk White adults** |
| --- | --- | --- |
| Non-obese | 1.69% | 3.53% |
| Overweight | 1.80% | 3.76% |
| Obesity Class I | 2.03% | 4.24% |
| Obesity Class II | 2.61% | 5.45% |
| Obesity Class III.I | 3.13% | 6.54% |
| Obesity Class III.II | 3.65% | 7.63% |

*Black calculation*

The mortality estimates calculated above are further adjusted for the Black population. [Luo et al. (2022](https://www.ncbi.nlm.nih.gov/pmc/articles/PMC8752946/)) report that Black patients have a significantly higher mortality risk than White patients when accounting for race, age, and sex (HR 1.46) (18). While they find in a subsequent model that accounting for income and education lowers the HR to 1.05, our model is simulating the difference in mortality for all incomes and education levels across the races, therefore the HR of 1.46 is applied to the mortality rates. The mortality rates for White CVD-risk adults is then further multiplied by 1.46 to obtain for the 60-69 age group:

| **Obesity group** | **Mortality rate for CVD-risk White adults** | **Mortality rate for CVD-risk Black adults** |
| --- | --- | --- |
| Non-obese | 1.71% | 2.50% |
| Overweight | 1.86% | 2.72% |
| Obesity Class I | 2.30% | 3.36% |
| Obesity Class II | 3.03% | 4.42% |
| Obesity Class III.I | 3.89% | 5.68% |
| Obesity Class III.II | 4.74% | 6.93% |

and for the age group 70-84 years:

| **Obesity group** | **Mortality rate for CVD-risk White adults** | **Mortality rate for CVD-risk Black adults** |
| --- | --- | --- |
| Non-obese | 3.53% | 5.16% |
| Overweight | 3.76% | 5.49% |
| Obesity Class I | 4.24% | 6.19% |
| Obesity Class II | 5.45% | 7.96% |
| Obesity Class III.I | 6.54% | 9.55% |
| Obesity Class III.II | 7.63% | 11.14% |

***Costs***

Semaglutide

Both White and Black populations are assumed to pay the newly negotiated price of semaglutide, $245 per month, even during the dose escalation period. Due to the use of ITT results for effectiveness and utility, we incorporate the dropout rate for the costs only. We assume patients have a constantly increasing dropout rate for the first 12 months of treatment ((18.9%/12 = 1.575%)*month), and after 12 months, all remaining patients continue with semaglutide. Expected costs are calculated as follows: E[cost] = (245+health state costs)*(1-dropout rate) + (health state costs)*(dropout rate) = health state costs + (245)*(1-dropout rate).

The patent for branded Wegovy® (which is currently approved under Medicare) is set to expire in 2032. Since our model spans a 25-year time horizon, we set a price reduction after 66 months or approximately 5.5 years (assuming the model begins July 1, 2026). Using information from an FDA report, we set a price reduction of 27% for the base-case (19). Although the report finds an average price reduction of 54% when there are two primary competitors for weight loss drugs (Novo Nordisk and Eli Lily), federal drug price negotiations may distort future price changes. Given the drastic current reduction in price, we assume that the price would then only drop by half its expected amount when its patent expires (54%/2) = 27%.

LSG

Both White and Black populations are assumed to pay the same price of surgery and early/late major/minor complications. However, the expected total cost of surgery does differ by race due to the higher rate of complications seen in the Black population. Expected costs of surgery are calculated as follows: E[surgery cost] =

Initial price + probability of major complications*cost of major complications +

probability of minor complications * cost of minor complications, using the early/late complication rates listed above.

***Utility Values***

Age-specific utility values for each obesity state (non-obese, overweight, Obesity Class I, Obesity Class II, Obesity Class III.I, and Obesity Class III.II) are obtained from Alsumali et al. (2018) (14). The article gives utility values for ages 61-70 and ages ≥ 71. Since the model covers both these age groups over the 25-year time horizon, the average utility value for each health state is used. Alsumali et al. (2018) do not differentiate between non-obese and overweight utility values, so the same utility value is assumed for both groups. Utility is assumed to not differ by race. Death has a utility value of 0. The utility values given by Alsumali et al. (2018) are listed below:

| **Health state** | **Ages 61-70 Utility** | **Ages ≥ 71 Utility** |
| --- | --- | --- |
| Non-obese | 0.81 | 0.79 |
| Obesity Class I | 0.79 | 0.76 |
| Obesity Class II | 0.76 | 0.74 |
| Obesity Class III.I | 0.71 | 0.69 |
| Obesity Class III.II | 0.66 | 0.66 |

and the final utility values used in the model are:

| **Health State** | **Utility** |
| --- | --- |
| Non-obese | 0.80 |
| Overweight | 0.80 |
| Obesity Class I | 0.775 |
| Obesity Class II | 0.75 |
| Obesity Class III.I | 0.70 |
| Obesity Class III.II | 0.66 |
| Dead | 0 |

There is a disutility from LSG accounted for in the model. Initial surgery is assumed to have a disutility of -0.22 lasting 2 weeks and minor and major complications have disutilities (lasting 2 weeks) of -0.11 and -0.36, respectively. These values are obtained from Campbell et al. (2010), and are assumed to apply to LSG (though they are for Laparoscopic Roux-en-Y gastric bypass surgery (LRYGB) surgery) (5). Thus, for example, expected utility in the first month after surgery for the White population is calculated as follows:

[Health state population vector]*[Health state utility values] + -0.22*(2/4.35) + 0.0540(-0.11*(2/4.35)+ 0.0297(-0.36*(2/4.35)

Recall that the initial surgery disutility and complications are multiplied by (2/4.35) to reflect that they last approximately 2 weeks within the 4.35 weeks that make up one month. And, recall that for the White population, the rate of early minor complications is 5.40% and the rate of early major complications is 2.97%.

The utilities for the Black population are calculated in a similar fashion but using complication rates for the Black population.

***Assuming Ranges***

Several parameters from the literature do not have ranges associated with them. Following precedent from Haseeb et al. (2024), we assume that if a range is not given, costs have a range of +/- 25% and all other parameters have a range of +/- 10% (20).

**Supplementary Tables and Figures**

**Table S1: Full Parameter List**

| **Parameter** | **White Base Case** | **White Range** | **Black Base Case** | **Black Range** | **Distribution** | **Source(s)** |
| --- | --- | --- | --- | --- | --- | --- |
| **Weight Trajectories** | | | | | | |
| ‘Do nothing’ change in BMI /month | 0.024 | 0, 0.0264 (+ 10%) | 0.024 | 0, 0.0264 | Normal | Zajacova et al. 2017 |
| Semaglutide change in  BMI /month (Y1) | -0.26 | -0.24, -0.28 | -0.26 | -0.24, -0.28 | Normal | Wilding et al. 2021; Ryan et al. 2024 |
| LSG change in BMI/month (Months 0-6) | -1.62 | -1.30, -1.93 | -1.36 | -1.09, -1.62 | Normal | Salminen et al. 2022; [Fernández-Ananín](https://www.zotero.org/google-docs/?UCdyQp) et al. 2022; Wood et al. 2019 |
| LSG change in BMI/month (Months 6-12) | -0.20 | -0.52, 0.13 | -0.17 | -0.44, 0.11 | Normal | Salminen et al. 2022; [Fernández-Ananín](https://www.zotero.org/google-docs/?UCdyQp) et al. 2022; Wood et al. 2019 |
| LSG change in BMI/month (Y2-Y5) | 0.04 | -0.004, 0.09 | 0.04 | -0.004, 0.09 | Normal | Salminen et al. 2022; [Fernández-Ananín](https://www.zotero.org/google-docs/?UCdyQp) et al. 2022 |
| LSG change in in BMI/month (Y6-end) | 0.02 | -0.02, 0.06 | 0.02 | -0.02, 0.06 | Normal | Salminen et al. 2022; [Fernández-Ananín](https://www.zotero.org/google-docs/?UCdyQp) et al. 2022 |
| **Complications** | | | | | | |
| Semaglutide Dropout Rate (%) | 18.9 | 17.01, 20.79  (+/- 10%) | 18.9 | 17.01, 20.79  (+/- 10%) | Beta | Wilding et al. 2021 |
| Semaglutide minor side effects (%) | 44.2 | 39.78, 48.62  (+/- 10%) | 44.2 | 39.78, 48.62  (+/- 10%) | Beta | Wilding et al. 2021 |
| LSG early minor complications (%) | 5.40 | 0.96, 11.30 | 7.06 | 1.27, 14.49 | Beta | Alsumali et al. 2018, Wood et al. 2019 |
| LSG early major complications (%) | 2.97 | 0.81, 8.82 | 3.88 | 1.07, 11.32 | Beta | Alsumali et al. 2018, Wood et al. 2019 |
| LSG late minor complications (%) | 0.39 | 0.26, 1.01 | 0.51 | 0.34, 1.29 | Beta | Alsumali et al. 2018, Wood et al. 2019 |
| LSG late major complications (%) | 0.09 | 0.04, 0.41 | 0.12 | 0.06, 0.53 | Beta | Alsumali et al. 2018, Wood et al. 2019 |
| **Costs** | | | | | | |
| **Non-treatment specific costs** | | | | | | |
| Non-obese | 4,529.49 | 3,397.12,  5,661.87  (+/- 25%) | 4,529.49 | 3,397.12,  5,661.87  (+/- 25%) | Gamma | Campbell et al. 2010 |
| Overweight | 4,529.49 | 3,397.12,  5,661.87  (+/- 25%) | 4,529.49 | 3,397.12,  5,661.87  (+/- 25%) | Gamma | Campbell et al. 2010 |
| Obesity Class I | 5,325.71 | 3,994.29,  6,657.14  (+/- 25%) | 5,325.71 | 3,994.29,  6,657.14  (+/- 25%) | Gamma | Campbell et al. 2010 |
| Obesity Class II | 6,265.80 | 4,699.35,  7,832.25  (+/- 25%) | 6,265.80 | 4,699.35,  7,832.25  (+/- 25%) | Gamma | Campbell et al. 2010 |
| Obesity Class III.I | 7,633.19 | 5,724.89,  9,541.49  (+/- 25%) | 7,633.19 | 5,724.89,  9,541.49  (+/- 25%) | Gamma | Campbell et al. 2010 |
| Obesity Class III.II | 9,457.81 | 7,093.36,  11,822.26  (+/- 25%) | 9,457.81 | 7,093.36,  11,822.26  (+/- 25%) | Gamma | Campbell et al. 2010 |
| **Treatment specific costs** | | | | | | |
| Semaglutide monthly cost ($) | 245 | 150, 649.42 | 245 | 150, 649.42 | NA | White House 2025, CMS 2026, CMS 2024 |
| Semaglutide price reduction (%) | 27 | 20.25, 33.75  (+/- 25%) | 27 | 20.25, 33.75  (+/- 25%) | Gamma | FDA 2019 |
| LSG initial cost ($) | 28,298.57 | 21,223.93,  35,373.21  (+/- 25%) | 28,298.57 | 21,223.93,  35,373.21  (+/- 25%) | Gamma | Alsumali et al. 2018 |
| LSG early minor complications cost ($) | 1,630.34 | 1,222.75, 2,037.92  (+/- 25%) | 1,630.34 | 1,222.75, 2,037.92  (+/- 25%) | Gamma | Alsumali et al. 2018 |
| LSG early major complications cost ($) | 53,159.74 | 39,869.80,  66,449.67  (+/- 25%) | 53,159.74 | 39,869.80,  66,449.67  (+/- 25%) | Gamma | Alsumali et al. 2018 |
| LSG late minor complications cost ($) | 1,021.74 | 766.30,  1,277.17  (+/- 25%) | 1,021.74 | 766.30,  1,277.17  (+/- 25%) | Gamma | Alsumali et al. 2018 |
| LSG late major complications cost ($) | 58,530.60 | 43,897.95,  73,163.25  (+/- 25%) | 58,530.60 | 43,897.95,  73,163.25  (+/- 25%) | Gamma | Alsumali et al. 2018 |
| **Utility** | | | | | | |
| Non-obese utility | 0.80 | 0.72,  0.88  (+/- 10%) | 0.80 | 0.72,  0.88  (+/- 10%) | Beta | Alsumali et al. 2018 |
| Overweight utility | 0.80 | 0.72,  0.88  (+/- 10%) | 0.80 | 0.72,  0.88  (+/- 10%) | Beta | Alsumali et al. 2018 |
| Obesity Class I utility | 0.775 | 0.6975,  0.8525  (+/- 10%) | 0.775 | 0.6975,  0.8525  (+/- 10%) | Beta | Alsumali et al. 2018 |
| Obesity Class II utility | 0.75 | 0.675,  0.825  (+/- 10%) | 0.75 | 0.675,  0.825  (+/- 10%) | Beta | Alsumali et al. 2018 |
| Obesity Class III.I utility | 0.70 | 0.63,  0.77  (+/- 10%) | 0.70 | 0.63,  0.77  (+/- 10%) | Beta | Alsumali et al. 2018 |
| Obesity Class III.II utility | 0.66 | 0.594,  0.726  (+/- 10%) | 0.66 | 0.594,  0.726  (+/- 10%) | Beta | Alsumali et al. 2018 |
| Semaglutide minor complications disutility | -0.02 | 0, -0.04 | -0.02 | 0, -0.04 | Beta | Pollack et al. 2010 |
| Initial surgery disutility | -0.22 | -0.24, -0.20 | -0.22 | -0.24, -0.20 | Beta | Campbell et al. 2010; Haseeb et al. 2024 |
| Minor complications disutility | -0.11 | -0.12, -0.10 | -0.11 | -0.12, -0.10 | Beta | Campbell et al. 2010; Haseeb et al. 2024 |
| Major complications disutility | -0.36 | -0.40, -0.32 | -0.36 | -0.40, -0.32 | Beta | Campbell et al. 2010; Haseeb et al. 2024 |
| **Mortality** | | | | | | |
| Non-obese mortality rate (%)-Age 60-69 years | 1.71 | 1.31, 1.89 | 2.50 | 1.92, 2.75 | Beta | Gonzalez et al. 2011; Pandya et al. 2017; KFF; Luo et al. 2022 |
| Overweight mortality rate (%)-Age 60-69 years | 1.86 | 1.42, 2.05 | 2.72 | 2.08, 2.99 | Beta | Gonzalez et al. 2011; Pandya et al. 2017; KFF; Luo et al. 2022 |
| Obesity Class I mortality rate (%)-Age 60-69 years | 2.30 | 1.76, 2.53 | 3.36 | 2.57, 3.69 | Beta | Gonzalez et al. 2011; Pandya et al. 2017; KFF; Luo et al. 2022 |
| Obesity Class II mortality rate (%)-Age 60-69 years | 3.03 | 2.32, 3.34 | 4.42 | 3.39, 4.87 | Beta | Gonzalez et al. 2011; Pandya et al. 2017; KFF; Luo et al. 2022 |
| Obesity Class III.I mortality rate (%)-Age 60-69 years | 3.89 | 2.98, 4.28 | 5.68 | 4.34, 6.25 | Beta | Gonzalez et al. 2011; Pandya et al. 2017; KFF; Luo et al. 2022 |
| Obesity Class III.II mortality (%)-Age 60-69 years | 4.74 | 3.63, 5.22 | 6.93 | 5.30, 7.62 | Beta | Gonzalez et al. 2011; Pandya et al. 2017; KFF; Luo et al. 2022 |
| Non-obese mortality rate (%)-Age 70-84 years | 3.53 | 2.70, 3.89 | 5.16 | 3.95, 5.68 | Beta | Gonzalez et al. 2011; Pandya et al. 2017; KFF; Luo et al. 2022 |
| Overweight mortality rate (%)-Age 70-84 years | 3.76 | 2.88, 4.14 | 5.49 | 4.20, 6.04 | Beta | Gonzalez et al. 2011; Pandya et al. 2017; KFF; Luo et al. 2022 |
| Obesity Class I mortality rate (%)-Age 70-84 years | 4.24 | 3.25, 4.67 | 6.19 | 4.74, 6.82 | Beta | Gonzalez et al. 2011; Pandya et al. 2017; KFF; Luo et al. 2022 |
| Obesity Class II mortality rate (%)-Age 70-84 years | 5.45 | 4.18, 6.00 | 7.96 | 6.10, 8.76 | Beta | Gonzalez et al. 2011; Pandya et al. 2017; KFF; Luo et al. 2022 |
| Obesity Class III.I mortality rate (%)-Age 70-84 years | 6.54 | 5.01, 7.20 | 9.55 | 7.31, 10.51 | Beta | Gonzalez et al. 2011; Pandya et al. 2017; KFF; Luo et al. 2022 |
| Obesity Class III.II mortality (%)-Age 70-84 years | 7.63 | 5.84, 8.40 | 11.14 | 8.53, 12.26 | Beta | Gonzalez et al. 2011; Pandya et al. 2017; KFF; Luo et al. 2022 |

**Figures S1-S3: One-way sensitivity analysis results.^*^**

**S1**

**S2**

**S3**

^*^In a one-way sensitivity analysis, comparisons may extend into negative ICER values, so population Net Health Benefit (NHB) values are used on the X-axis instead.

**Figure S4: PSA results for Black and White populations**

**Figure S5: CEAC Results with Previous Price of Semaglutide (Net Price= $649.42)**

**Figure S6: CEAC Results with Lowest Price of Semaglutide ($150)**

**Figures S7-S9: Equity Results and Calculations**

**Figure S7: Step 1—Calculate the Incremental Net Health Benefit (iNHB) under the (a) Zero Payment assumption and (b) Proportional Payment assumption**

**Figure S8: Step 2–Use the calculated iNHB to adjust the QALE for both groups**

**Figure S9: Step 3–Calculate the difference in QALE for both groups. Under both the zero payment and proportional payment assumptions, semaglutide is the more equitable intervention.**

**‘**

**Figure S10: Heatmap of semaglutide price against increasing inequality aversion parameters (ε).**

Red and orange areas indicate where LSG is the preferred intervention and yellow and green areas indicate where semaglutide is the preferred intervention. The solid black line indicates the price threshold under which semaglutide is the preferred intervention. The dotted black line indicates the current price of semaglutide ($245). The square and triangle points indicate our assumed ε range of 0.5-3.0.

**Figure S11: Heatmap of additional lifetime QALYs against increasing inequality aversion parameters (ε).**

Yellow area indicates where LSG is the preferred intervention and orange and purple areas indicate where semaglutide is the preferred intervention. The black line indicates the additional QALYs threshold over which semaglutide is the preferred intervention. The square and triangle points indicate our assumed ε range of 0.5-3.0.

**Table S2: Scenario analysis results**

| **Race-Specific Parameter** | **Original NHB Value for the Black population** | **New NHB Value** |
| --- | --- | --- |
| LSG monthly weight loss (Months 1-6) | -940 QALYs | 1495 QALYs |
| LSG monthly weight loss (Months 6-12) | -940 QALYs | -565 QALYs |
| LSG minor complications (late) | -940 QALYs | -742 QALYs |
| LSG minor complications (late) | -940 QALYs | -866 QALYs |
| LSG major complications (early) | -940 QALYs | -871 QALYs |
| LSG minor complications (early) | -940 QALYs | -935 QALYs |
| Health-State Mortality Rates | -940 QALYs | -1534 QALYs |
| Population distribution among obesity classes | -940 QALYs | -6039 QALYs |

White-specific parameter estimates were substituted in the Black population model. Results are sorted by parameter that closes the outcome gap most between the Black NHB (-940 QALYs) and the White NHB (9,542 QALYs).

**References**

1.	Juliette Cubanski, Tricia Neuman, Nolan Sroczynski, Anthony Damico. A New Use for Wegovy Opens the Door to Medicare Coverage for Millions of People with Obesity [Internet]. KFF; 2024 Apr. Available from: https://www.kff.org/medicare/issue-brief/a-new-use-for-wegovy-opens-the-door-to-medicare-coverage-for-millions-of-people-with-obesity/

2.	Distribution of People with Medicare by Race/Ethnicity [Internet]. KFF. Available from: https://www.kff.org/medicare/state-indicator/people-with-medicare-by-raceethnicity/

3.	Medicare Beneficiaries by Age [Internet]. KFF; 2024. Available from: https://www.kff.org/medicare/state-indicator/medicare-beneficiaries-by-age/?currentTimeframe=0&sortModel=%7B%22colId%22:%22Location%22,%22sort%22:%22asc%22%7D

4.	Suehs BT, Kamble P, Huang J, Hammer M, Bouchard J, Costantino ME, et al. Association of obesity with healthcare utilization and costs in a Medicare population. Curr Med Res Opin. 2017 Dec;33(12):2173–80. doi:10.1080/03007995.2017.1361915 PubMed PMID: 28760001.

5.	Campbell J, McGarry LA, Shikora SA, Hale BC, Lee JT, Weinstein MC. Cost-effectiveness of laparoscopic gastric banding and bypass for morbid obesity. Am J Manag Care. 2010 Jul 1;16(7):e174-187. PubMed PMID: 20645663.

6.	Zajacova A, Huzurbazar S, Greenwood M, Nguyen H. Long-Term BMI Trajectories and Health in Older Adults: Hierarchical Clustering of Functional Curves. J Aging Health. 2015 Dec;27(8):1443–61. doi:10.1177/0898264315584329 PubMed PMID: 25953813; PubMed Central PMCID: PMC5436799.

7.	Alexander GC, Xiao X, Dilek S, Lewis S, Deng Q, Kim M, et al. Heterogeneity of Treatment Effects of Glucagon-Like Peptide-1 Receptor Agonists for Weight Loss in Adults: A Systematic Review and Meta-Analysis. JAMA Intern Med. 2026 May 1;186(5):567–77. doi:10.1001/jamainternmed.2025.8222

8.	Wilding JPH, Batterham RL, Calanna S, Davies M, Van Gaal LF, Lingvay I, et al. Once-Weekly Semaglutide in Adults with Overweight or Obesity. N Engl J Med. 2021 Mar 18;384(11):989–1002. doi:10.1056/NEJMoa2032183 PubMed PMID: 33567185.

9.	Ryan DH, Lingvay I, Deanfield J, Kahn SE, Barros E, Burguera B, et al. Long-term weight loss effects of semaglutide in obesity without diabetes in the SELECT trial. Nat Med. 2024 Jul 1;30(7):2049–57. doi:10.1038/s41591-024-02996-7

10.	Salminen P, Grönroos S, Helmiö M, Hurme S, Juuti A, Juusela R, et al. Effect of Laparoscopic Sleeve Gastrectomy vs Roux-en-Y Gastric Bypass on Weight Loss, Comorbidities, and Reflux at 10 Years in Adult Patients With Obesity: The SLEEVEPASS Randomized Clinical Trial. JAMA Surg. 2022 Aug 1;157(8):656–66. doi:10.1001/jamasurg.2022.2229

11.	Fernández-Ananín S, Ballester E, Gonzalo B, Codina C, Miñambres I, Pérez A, et al. Is Sleeve Gastrectomy as Effective in Older Patients as in Younger Patients? A Comparative Analysis of Weight Loss, Related Comorbidities, and Medication Requirements. Obes Surg. 2022 Jun;32(6):1909–17. doi:10.1007/s11695-022-05940-1 PubMed PMID: 35411452; PubMed Central PMCID: PMC9072441.

12.	Wood MH, Carlin AM, Ghaferi AA, Varban OA, Hawasli A, Bonham AJ, et al. Association of Race With Bariatric Surgery Outcomes. JAMA Surg. 2019 May 15;154(5):e190029–e190029. doi:10.1001/jamasurg.2019.0029

13.	Pollack MF, Purayidathil FW, Bolge SC, Williams SA. Patient-reported tolerability issues with oral antidiabetic agents: Associations with adherence; treatment satisfaction and health-related quality of life. Diabetes Res Clin Pract. 2010 Feb 1;87(2):204–10. doi:10.1016/j.diabres.2009.11.023

14.	Alsumali A, Eguale T, Bairdain S, Samnaliev M. Cost-Effectiveness Analysis of Bariatric Surgery for Morbid Obesity. Obes Surg. 2018 Aug 1;28(8):2203–14. doi:10.1007/s11695-017-3100-0

15.	Berrington de Gonzalez A, Hartge P, Cerhan JR, Flint AJ, Hannan L, MacInnis RJ, et al. Body-mass index and mortality among 1.46 million white adults. N Engl J Med. 2010 Dec 2;363(23):2211–9. doi:10.1056/NEJMoa1000367 PubMed PMID: 21121834; PubMed Central PMCID: PMC3066051.

16.	Pandya A, Sy S, Cho S, Alam S, Weinstein MC, Gaziano TA. Validation of a Cardiovascular Disease Policy Microsimulation Model Using Both Survival and Receiver Operating Characteristic Curves. Med Decis Mak Int J Soc Med Decis Mak. 2017 Oct;37(7):802–14. doi:10.1177/0272989X17706081 PubMed PMID: 28490271; PubMed Central PMCID: PMC5577377.

17.	Meredith Freed, Juliette Cubanski, Michelle Long, Nancy Ochieng, Tricia Neuman, Alina Salganicoff. 10 Key Facts About Women with Medicare. KFF [Internet]. 2024 Apr 30. Available from: https://www.kff.org/medicare/issue-brief/10-key-facts-about-women-with-medicare/#:~:text=In%202021%2C%20more%20than%20half,85%20and%20older%20(12%25).

18.	Luo J, Hendryx M, Wang F. Mortality disparities between Black and White Americans mediated by income and health behaviors. SSM - Popul Health. 2022 Mar;17:101019. doi:10.1016/j.ssmph.2021.101019 PubMed PMID: 35036515; PubMed Central PMCID: PMC8752946.

19.	Ryan Conrad, Randall Lutter. Generic Competition and Drug Prices: New Evidence Linking Greater Generic Competition and Lower Generic Drug Prices [Internet]. Food and Drug Administration; 2019. Available from: https://www.fda.gov/media/133509/download

20.	Haseeb M, Chhatwal J, Xiao J, Jirapinyo P, Thompson CC. Semaglutide vs Endoscopic Sleeve Gastroplasty for Weight Loss. JAMA Netw Open. 2024 Apr 12;7(4):e246221–e246221. doi:10.1001/jamanetworkopen.2024.6221
